# The Silent Pandemic: Why COVID-19-Induced Loneliness Is No Laughing Matter

**DOI:** 10.1017/dmp.2021.14

**Published:** 2021-01-08

**Authors:** Matan Bone, Manavi Purohit, Moska Sial, Kishan Pankhania, Abdulaziz Alkhayyat

**Affiliations:** Faculty of Biology, Medicine and Health, The University of Manchester, Manchester, United Kingdom

**Keywords:** government, health policy, pandemics, policy making, quarantine

The 2019 coronavirus disease (COVID-19) pandemic is wreaking havoc across societies. The actions taken by countries to contain the disease have resulted in population-wide social and psychological challenges. The elderly is arguably the most vulnerable group, susceptible to both physical disease complications and the social implications levied by the pandemic.

Policy-makers have constructed plans in response to the COVID-19 pandemic, targeting physical health hazards, especially in hospitals and care homes. Yet, an area overlooked is loneliness among high-risk groups, like the elderly. In the work of van Tilburg et al.,^1^ among 1679 elderly Dutch citizens, there is a 34% increase in the perception of loneliness and a 16% increase in feelings of emptiness between October 2019 (pre-pandemic) and May 2020. The psychological implications of the pandemic for the elderly are often understated and are significant determinants of well-being in this cohort. Ignoring the effects of loneliness of the elderly carries repercussions, as psychological hardships often manifest as physical pathology. Raheel et al.^2^ correlates loneliness with an increased incidence of neurocognitive, immunological, and endocrine disorders.

While the literature affirms the importance of psychological care in the elderly, a precise framework for the implementation of care is rarely provided.^1-3^ To properly appreciate its magnitude and severity and deduce proper plans to tackle it, COVID-19-induced loneliness must be recognized as a pandemic. A pandemic is defined as “an epidemic occurring worldwide, or over a very wide area, crossing international boundaries and usually affecting a large number of people,”^4^ and COVID-19-induced loneliness fulfills this definition. Such recognition would highlight the importance of the problem and ensure that sufficient resources are provided to tackle it. Any plan must also recognize high-risk groups, like the elderly, who require more support. Residential and long-term care facilities must be provided with frameworks for the implementation of governmental policies. One proposal put forward by Zhang et al.^5^ offers an approach that encompasses psychological intervention both during and after a health crisis, suggesting that it ought to constitute an integrated part of the treatment of pneumonia. The plan must provide caregivers with a cognitive-behavioral model to target loneliness. Van Orden et al.^3^ suggests that a model should provide the elderly with coping strategies to manage social isolation. This approach ought to enhance social connectedness by providing the elderly with new opportunities to connect to family and friends and by teaching them how to alter their perspective and obtain a more optimistic outlook on the situation.^3^

Amidst a second wave of infections, it is critical to appreciate the necessity of mental-health care provision focusing on loneliness in the elderly. Comprehensive guidelines are encouraged to prepare residential and long-term care facilities to recognize and address loneliness. While the end of the physical pandemic is in the foreseeable future, the mental-health pandemic will continue to mount casualties for years unless significant steps are undertaken. The dogma must be clear: loneliness is a pandemic; it is not less of a public-health hazard than COVID-19, and the victims of loneliness must not be forgotten.
